# Supplementary material for: Supporting disengaged children and young people living with diabetes to self-care: a qualitative study in a socially disadvantaged and ethnically diverse urban area
Source: BMJ Open. 2021 Oct 13;11(10):e046989. doi: 10.1136/bmjopen-2020-046989 (PMC8515452; doi:10.1136/bmjopen-2020-046989)
Supplement: Author's manuscript [file bmjopen-2020-046989.draft_revisions.pdf]

BMJ Open

BMJ Open is committed to open peer review. As part of this commitment we make the peer review history of every article we publish publicly available.

When an article is published we post the peer reviewers' comments and the authors' responses online. We also post the versions of the paper that were used during peer review. These are the versions that the peer review comments apply to.

The versions of the paper that follow are the versions that were submitted during the peer review process. They are not the versions of record or the final published versions. They should not be cited or distributed as the published version of this manuscript.

BMJ Open is an open access journal and the full, final, typeset and author-corrected version of record of the manuscript is available on our site with no access controls, subscription charges or pay-per-view fees (http://bmjopen.bmj.com).

If you have any questions on BMJ Open's open peer review process please email info.bmjopen@bmj.com

BMJ Open

Supporting disengaged children and young people living with diabetes to self-care: A qualitative study in socially disadvantaged and ethnically diverse urban areas

Journal:	*BMJ Open*
Manuscript ID	bmjopen-2020-046989
Article Type:	Original research
Date Submitted by the Author:	15-Nov-2020
Complete List of Authors:	Sharpe, Darren; University of East London, Institute for Connected Communities (ICC)<br>Rajabi, Mohsen; University of Tehran<br>Harden, Angela; City University of London, Centre for Maternal and Child Health Research, School of Health Sciences<br>Moodambail, Abdul; Barts Health NHS Trust, Department of Paediatrics, Newham University Hospital<br>Hakeem, Vaseem ; Barnet and Chase Farm Hospitals NHS Trust, Royal Free London NHS Foundation Trust
Keywords:	DIABETES & ENDOCRINOLOGY, Change management < HEALTH SERVICES ADMINISTRATION & MANAGEMENT, Community child health < PAEDIATRICS, Quality in health care < HEALTH SERVICES ADMINISTRATION & MANAGEMENT

SCHOLARONE™
Manuscripts

I, the Submitting Author has the right to grant and does grant on behalf of all authors of the Work (as defined in the below author licence), an exclusive licence and/or a non-exclusive licence for contributions from authors who are: i) UK Crown employees; ii) where BMJ has agreed a CC-BY licence shall apply, and/or iii) in accordance with the terms applicable for US Federal Government officers or employees acting as part of their official duties; on a worldwide, perpetual, irrevocable, royalty-free basis to BMJ Publishing Group Ltd ("BMJ") its licensees and where the relevant Journal is co-owned by BMJ to the co-owners of the Journal, to publish the Work in this journal and any other BMJ products and to exploit all rights, as set out in our licence.

The Submitting Author accepts and understands that any supply made under these terms is made by BMJ to the Submitting Author unless you are acting as an employee on behalf of your employer or a postgraduate student of an affiliated institution which is paying any applicable article publishing charge ("APC") for Open Access articles. Where the Submitting Author wishes to make the Work available on an Open Access basis (and intends to pay the relevant APC), the terms of reuse of such Open Access shall be governed by a Creative Commons licence – details of these licences and which Creative Commons licence will apply to this Work are set out in our licence referred to above.

Other than as permitted in any relevant BMJ Author's Self Archiving Policies, I confirm this Work has not been accepted for publication elsewhere, is not being considered for publication elsewhere and does not duplicate material already published. I confirm all authors consent to publication of this Work and authorise the granting of this licence.

For peer review only

Supporting disengaged children and young people living with diabetes to self-care: A qualitative study in socially disadvantaged and ethnically diverse urban areas

Darren Miguel Sharpe [1*], Mohsen Rajabi[2], Angela Harden[3], Abdul Moodambail [4], Vaseem Hakeem [5]

Institute for Connected Communities, University of East London, London, UK. D.sharpe@uel.ac.uk

[2] Department of Psychology, University of Tehran, Tehran, Iran. Rajabimohsen@ut.ac.ir

ORCID: https://orcid.org/0000-0002-0254-9027

[3] Centre for Maternal and Child Health Research, School of Health Sciences, City, University of London, London, UK. A.harden@uel.ac.uk

[4] Clinical Lead in Paediatrics, Barts Health Trust, Newham University Hospital, London, UK. Abdul.Moodambail@bartshealth.nhs.uk

[5] Consultant Paediatrician, Barnet Hospital, Royal Free London NHS Foundation Trust, London, UK. Vaseem.hakeem@nhs.net

*Corresponding Author: Institute for Connected Communities, University of East London, Stratford Campus, Water Lane, London, E15 4LZ, UK.

Email address: D.sharpe@uel.ac.uk

ORCID: https://orcid.org/0000-0001-7418-4496

Telephone: +442082234325

Word Count:

For peer review only - http://bmjopen.bmj.com/site/about/guidelines.xhtml

3,963

Abstract

Objective. To explore how to enhance services to increase take-up by children and young people (CYP) clinically considered 'disengaged' by diabetes services.

Design, setting and participants. This is an applied qualitative research study conducted in an ethnically dense, socially disadvantaged area in London. The sample consisted of twenty-two participants who were recruited from two clinics (e.g. 0-18 and 16-25) and clinically categorised as 'disengaged'. The participants were predominately from white British, Eastern European or South Asian backgrounds and on average aged fifteen. The mean age of diagnoses was 9.5 years.

Results. The findings confirm the crucial importance of non-medicalised care in children and young people diabetes care. A life plan is considered as important to the CYP as a health plan. Participant's said that they valued the holistic support provided by friends, family members and school teachers but found structural barriers in their health and educational pathways as well as disparities in the quality of help and support provided to them at critical moments along the life course as they actively try to maximise their well-being by balancing their life priorities against their diabetes priorities. Combined these features undermine the participant's engagement in health services where personal strategies are often held back and/or edited out of clinical appointments out of fear of condemnation.

Conclusion. We demonstrate why diabetes health teams need to appreciate the conflicting pressures experienced by CYP and to co-produce more nuanced health plans for addressing their concerns regarding identity, risk taking behaviours in the context of their life-worlds. Exploring these issues and identifying ways to better support CYP more pro-actively should reduce perceived disengagement and set realistic health outcomes that make best use of medical resources.

Key words:

For peer review only - http://bmjopen.bmj.com/site/about/guidelines.xhtml

Disengagement, children and young people, diabetes services, self-care

Strengths and limitations of this study

- The study recruitment strategy paradoxically did not reach the hardest to reach groups of young patients.
- Child and parent joint interviews need careful thought and consideration to avoid replicating the power dynamics in clinical appointments.
- Child and parent joint interviews revealed how attitudes towards different treatment are transmitted across the family.
- The study conversation-discussion format successfully allowed young patients to talk in-depth on what matter most to them.
- The study scheduled interviews to fit around the lives of young people, which increased up-take.

For peer review only

For peer review only - http://bmjopen.bmj.com/site/about/guidelines.xhtml

INTRODUCTION

"Appointments can feel a bit intimidating – especially when there are five or six people in the room at the time. I do not like feeling judged or stared at. We should be able to have an open discussion about diabetes in relation to my risk-taking behaviour."

(Participant 17, aged 13)

The study has systematically collected and considered patient's perspective of the patient-doctor-family-school-peer relationship has part of diabetes self-care. Listening carefully to children and young people (CYP) who had all been categorised as becoming disengaged with their diabetes care teams, as enabled the research team to amplify their voices to help inform and influence commissioning guidance to improve diabetes services for CYP living in a poor and ethically dense communities in London. It is well-recognised that CYP with long-term conditions may disengage from clinical services during adolescence and emerging adulthood. For CYP this can often contribute to poor health outcomes and for services a waste of medical resources.

Diabetes self-care includes a range of activities (e.g., self-monitoring of blood glucose, eating a low-saturated-fat diet, and checking one's feet), and it is now well established that these different components do not correlate highly [1]. In spite of structured education programmes and regular health promotion messages made by health professionals, researchers and charities, we seldom hear the voices of CYP living with diabetes. We do not hear how they approach food and exercise, as well as navigating the physiological and psychological changes consistent with growing up, and how they go about having their voices heard in an NHS culture arguably dominated by its paternalism [2–5]. The experience of being 'silenced' can often be compounded by parents, who do not give

For peer review only - http://bmjopen.bmj.com/site/about/guidelines.xhtml

power away for putting their son's/daughter's medical care plan into practice. Thus, for CYP living with diabetes to take ownership for their self-care is far more complex than adherence to the health plan but must negotiate and navigate a number of different relationships and contexts.

Research highlights the negative long-term health outcomes for members of the public who disengage from health services [6–8]. According to NHS England [9] 'disengagement' is defined when a CYP, or carer does not respond to requests from health professionals. Behaviours of disengagement are usually cumulative and may include: disregarding health appointments; not having a GP; not being home for professional visits; not allowing professionals into the home; agreeing to take action but never doing it; hostile behaviour towards professionals; manipulative behaviour resulting in no health care; actively avoiding contact with professionals; and attendance at urgent care centres, accident and emergency departments but not waiting to be seen/taking own discharge [9]. Elders [10] argues that people most likely to disengage are characteristically, "young; are from more deprived areas; are more commonly anxious and depressed; have higher HbA1c values; and are more frequently male" (p115).

Much of the medical literature on non-attendance in diabetes points to significantly higher HbA1c results amongst so-called 'defaulters' used as an example of the benefits of clinic attendance [11]. In England, there is evidence that young people miss more scheduled medical appointments of all kinds than other age groups [12,13]. Indeed, for younger patients, the transition from paediatric to adult diabetes clinics, is highlighted as a critical moment when young people drop out of the system [14]. However, reviews of the existing literature do not offer conclusive reasons for why and show that clinic-related factors behind non-attendance are rarely assessed, from the young patients perspectives [15,16]. Freeborn [17] stresses that understanding the challenges of youth living with diabetes, from their perspective, is the first step in improving diabetes outcomes for this age group.

For peer review only - http://bmjopen.bmj.com/site/about/guidelines.xhtml

This study focuses on this known challenge and problematise the notion of 'disengagement', from the patient point of view. We show how CYP living with diabetes often navigate and negotiate conflicting social pressures to build competencies and resilience, to form a stable sense of self and to achieve intimacy through relationships in spite of living with diabetes.

This paper argues for non-medicalised self-care to gain parity with medicalised forms. In other words, a health plan is considered as important to the CYP as a life plan. We explore how both medicalised and non-medicalised forms of self-care often intersect in the daily lives of ethnically diverse CYP living in disadvantaged areas, and what this means for health care professionals.

METHODS

This was a qualitative study undertaken in May 2016 to May 2018. We undertook in-depth qualitative interviews with twenty-two CYP (aged 10 to 25) to assess their understanding and meaning of attending clinics and living with diabetes. The study was led by a team of adult researchers with the help of young co-investigators with Type 1 and 2 diabetes. The interviews were also supplemented by a systematic literature review and inquiry workshops with stakeholders. All steps were followed by the SRQR guideline [18].

Recruitment

We used a non-probability sampling strategy to identify and recruit CYP from two clinical registers who had been categorised as 'disengaged' by their clinician as per the NHS Policy of Engagement and Disengagement with services definition [9]. The baseline demographics of participant's shows that they were predominately white British, Eastern European or South Asian and aged between 11 and 19 with diabetes types 1 and 2 (Supplementary File). Potential participants were first approached by a member of the

For peer review only - http://bmjopen.bmj.com/site/about/guidelines.xhtml

health care team, followed by a screening call from the research team leading often to an interview.

For peer review only

Data analysis

Interviews were digitally recorded and transcribed, and underwent content analysis to generate themes [19]. This involved repeated readings of the transcripts to gain familiarity with the content, the use of coding to identify recurring, similar and contrasting content, and the collapsing of codes into central themes.

Ethics

Ethical approval was obtained by the local NHS ethic committee in combination with the University of East London ethics committee. All the participants' (and parents) provided written consent or assent and all names have been pseudonymised.

Results

This section illustrates where and how participants negotiate and navigate the pressures of health care service, education, home and social networks as part of their diabetes self-care.

Stigmatized status

Participants' shared common stories of how their peer groups behave, look, and how they think. Participant's normative notions of peer norms revealed more about how they positioned themselves at the centre and/or margins of such norms, than what it might reveal about the normative perceptions and behaviour of their peers. Participants said:

> If I was to walk on the street, no one could actually tell that I had diabetes, unless I actually do something … for example a blood test… (P7, aged 15).

> Now at age 15, I have realised that diabetes doesn't make me different from anybody else (P10, aged 15).

For peer review only - http://bmjopen.bmj.com/site/about/guidelines.xhtml

For peer review only

The relationship with self and others is complex and can positively and negatively impact on self-care. One participant explains:

> I was sixteen and, on the bus, and I just didn't feel well, and I did my injection and the woman [passenger] said, "oh my god", and I was, like, sorry yeah? And they're like, "you know it's not good to take drugs on the bus?" And I'm like, "excuse me", and my friend just started laughing like. Cos we couldn't believe it and I'm like "it's not drugs. I'm diabetic" (P16, aged 17).

Age was not the only marker of difference. Participants identified as belonging to ethnic minority groups (or linguistic groups), overlaid with intra group differences based on their gender, as well as lifestyle differences bought upon by living with diabetes. Our analysis has shown that the perception of social stigma (e.g. disapproval of a person based on socially characteristic grounds that are perceived) is a common feature negotiated in how participants experience building a stable social identity [20,21] whilst managing a chronic disease [22,23].

The need to belong often undermines the participants' medicalised care practices in order to minimise their difference and to increase the likelihood of inclusion by peers and in wider society. Paradoxically, participants reported feeling a greater sense of wellbeing, feeling safe and feeling valued in ethnically and/or religiously defined spaces they inhabited than inside the clinic room, where they felt scrutinised and compartmentalised.

Faith-based identity

Participants identified as both Christian and Muslim (Supplementary File) reported their belief system played a significant role in their self-care, which have had positive and negative consequences for their medical care. For example, participants gave accounts where religious observance helped them with their self-care. One participant said:

For peer review only - http://bmjopen.bmj.com/site/about/guidelines.xhtml

> As a Muslim you can't drink, [and I] don't smoke anyway, but there are rules like that, and, like, I do go out with friends but it's [drinking and smoking], not something I majorly think about (P13, aged 18).

In contrast, two participants said:

> [Ramadan] is a sacred month for us. So, yeah, I want to be part of that sacred thing as well, so that's why I feel upset when I can't do it as well (P7, aged 15).

> I had a DKA [diabetic ketoacidosis] over Ramadan. My mother was away and I was at home with my older sister. I wanted to experience fasting and the feast at the end. However, I ended up in hospital (P11, aged 18).

Central to most, but not all, the participants' accounts are the importance they've attached to their faith-based identities and how normative practices shared across their faith communities have been performed and, on occasion, have resulted in positive and negative self-care. Ramadan is an exemplifier of one such practice that can serve to resources identity but can also undermine healthy behaviours, so too are many other festivities and cultural events that requires a break from the routine nutritional diet. The clinical team should acknowledge the cultural spaces in which CYP inhabit and expect deviations from the idealized medical plans.

Supporting a CYP with diabetes in education

In the participants accounts of performing self-care their educational experiences were central features, with a specific focus on how teachers act as proxy health care workers. Participants said;

For peer review only - http://bmjopen.bmj.com/site/about/guidelines.xhtml

…my tutor helps me a lot cos I have to test at the right time, and sometimes I forget, so my tutor is there to help me if I'm doing it right or I'm doing it wrong (P3, aged 11).

At school I use, like, a special room. I would disappear into it [to do injections]. (P9, aged 15)

They [school] have an insulin register. If anybody is missing on the register, they go and look for them and remind them, have you eaten? (P7, aged 15).

Participants highlighted how policies and procedures in high-poverty schools, have helped to support them in their self-care. However, participants' accounts show challenges to self-care when transitioning between schools and then onto college and University. Participants mentioned:

Going from primary to secondary school and then college is difficult in relation to the information and knowledge they [educators] have about my diabetes. …you receive less and less help and therefore educators know less and less about diabetes. (P11, aged 18)

I experienced problems moving away from home to university and trying to sort out my studies, prescriptions and doctor appointments. (P2, aged 18)

Participants highlighted the positive and negative features of school-based diabetes support [24], with teachers playing a crucial role [25]. However, participants also observed a lack of training opportunities in schools to help teachers to improve their knowledge of diabetes care [26–29]. The overly reported challenge has been in transitions, when ironically support tends to tail off [30]. What is missing is a coordinated approach led by the health team in preventing the marginalisation of the CYP health plan, especially during stressful life events in their educational journeys, which result in high or imbalanced sugar

For peer review only - http://bmjopen.bmj.com/site/about/guidelines.xhtml

levels leading to hospitalisation. In practice, participants have tended to figure things out for themselves when the health care team should help to produce a smooth transition.

Friendship networks, and not the health care team - formed a conceptual bridge between home and school, which was felt to be especially important to CYP with diabetes [31,32]. Peers are a central factor in a child's socialization whether they have diabetes or not, and also highlight the fact that there are few studies addressing the role of friends among CYP in the management of the disease. A selection of accounts illustrates the type of support provided by friends in their self-care. Participants said:

> I collapsed in the middle of the playground and he [best friend] was taught by my mum what to do. So, then he called my mum, who told him to call 999. (P7, aged 15)

> …when I know I'm going out for a drink with friends, I make sure that I have my meal and my sugar levels are good. When you're drinking, obviously I don't get in to a state where I don't know what I'm doing like. I'm scared to be, like, overly drunk and waking up in a diabetic coma or something. (P19, aged 18)

Participants' accounts reveal how they use friendship networks as a source of support in their self-care, as argued by Salamon [33]. Yet still, social isolation and loneliness effects CYP and a few of the participant's experienced weak friendship networks and did not know other CYP in their cohort living with diabetes to relate too. Participants remark:

> I think as a child you want to really open up and talk to someone that understands what you're going through, understands the injections, and the needles. I think when I first got diabetes, I didn't know anyone with it so your kind of like, you can't talk about it with anybody. (P16, aged 17)

> I guess I don't get any real support. I have a couple of friends on Facebook, and we basically help each other through anything. (P10, aged 15)

For peer review only - http://bmjopen.bmj.com/site/about/guidelines.xhtml

On the surface, health care teams appear absent in helping to educate friends or lack knowledge of the support provided by friends for CYP in their care. The home environment has been important to understanding how participants make decisions affecting their self-care. The study takes has given the emotional consequences for CYP living in families undergoing sustained economic strain. CYP living in poverty are more likely to feel like a failure and have a sense of hopeless about their future than their more affluent peers. As a result, participants' choices around their self-care have often been made against the backdrop of epistemic health inequalities. In the face of economic hardships participants provide rich descriptions in how family members often serve as a crucial source of support in their self-care. Participants said;

> I have lived with diabetes for the last thirteen years. It hasn't always been under control. It was when my mother took responsibility for me that my diabetes was more controlled, more freedom means less control. (P11, aged 18)

> I feel like because they [older family members] were like born with it [diabetes], they don't really talk to us about it, you know, how it's affected them or what they do. (P4, aged 12)

> I go to the gym with my auntie. It's like a peace of mind away from home. They have the little TV screens in front of each activity and on the cross trainer and the treadmill, and on the steps and things. So, I can just watch TV (P8, aged 18).

Seldom do studies capture the lives of CYP as they are lived in real time, especially from ethnic minority and economically disadvantaged communities. Evidence suggests that conscientiously caring for the carer helps CYP to reach their full potential in increasing resilience living with diabetes. This is reflected in: psychological models of assessment of care givers; understanding illness belief systems [34]; interdisciplinary working to help reduce family stress and anxiety [35]; including fathering a child with diabetes [36]. The

For peer review only - http://bmjopen.bmj.com/site/about/guidelines.xhtml

increasing diversity experienced in London necessitates the cultural competency of the health care workers into the needs of families living in economic stress in order to provide the right support at the right time to care givers.

Diabetes self-management

Too often, participants behaviour and attitudes have been wrongly characterised by clinicians has a marker of their disengagement from the service. The markers are hardly ever directly discussed by the health care team or voluntarily disclosed in clinical appointment by CYP but loom large over the relationship. Participants said:

> At first, I didn't really take it [diabetes] that seriously, I suppose, but then when you realise it's going to affect you every day, then I started to take it more seriously. (P9, aged 15)

> I try not to eat as much, so that my blood sugar level doesn't go so high and [it] saves me from having [to] reinject myself with more insulin. (P6, aged 13)

> I'll eat what I want, I'll drink what I want and that's when my sugar levels started running high and I was just uncontrolled. So, I was missing appointments sometimes, like, oh, I'm just going to miss, I don't want to go. (P2, aged 18)

> There are certain things you must cut back on. I used to do boxing, which was intense training, for an hour or so, so I think stuff like that I kind of left. I haven't been doing it as much. I feel it's quite a lot harder to do with diabetes. (P16, aged 17)

In the latter case, no clinical support was provided to the participant to learn how to cope with high intensity exercise, in order to relieve the anger that so many of participants felt living with the condition. We see missed opportunity by health care teams to educate and support CYP to build liveable lives. Also, participants, highlight the inherent complications in implementing their health plan. Participants said;

For peer review only - http://bmjopen.bmj.com/site/about/guidelines.xhtml

> I'm constantly having to remember to do my blood test, and do my injections. Plus, with me, when I do my injection, after doing it, it does hurt. I get a bit frustrated and you don't want to do it afterwards. (P4, aged 12)

> Most of the time, I find [doing injections] easy. Sometimes it's kind of annoys me that I must keep doing it and doing it, and sometimes it hurts, and, yeah, fear and perceptions of doing injections. (P2, aged 18)

> You can't really tell I'm wearing a pump right now because everything is electronic blue tooth? I know that the pump and the meter will connect, automatically vibrate, so I will know the insulin will get delivered. Whereas, with the pen, I have to pick up my clothes, to open this, put a needle in the pen, do this, it's like a big long procedure before I actually give myself the injection. (P7, aged 15)

Using technology in self-care is proposed to CYP as a way to promote better self-care; however, this benefit is not borne out in this study. Indeed, Balfe [29] highlights how little attention has been paid to the accounts of CYP of the reasons why they may experience difficulties with their diabetes technology. Participants said:

> I tried it [pump] for a while, but it's very painful. It can be an inconvenience practically. I was doing PE, for example, and I had it on, it would just be annoying because I couldn't play football or anything. (P20, aged 18)

> I'm getting use to putting my carb count in the machine so it does the maths for me. I'm not really good at that because I like doing it mentally and, like, turn off the machine straight away. (P7, aged 15)

The five principle circumstances or markers that, so far as we have been able to observe, that signal CYP disengagement from clinical services include: (1) the probability or improbability of achieving normalcy when confronted with social stigma; (2) the easiness

For peer review only - http://bmjopen.bmj.com/site/about/guidelines.xhtml

or difficulty in integrating faith-based practices into their daily routines; (3) the consistency or inconsistency in the quality of support provided in education; (4) the constancy or inconstancy of family and friends; and (5) the obstacles and practical challenges in balancing medicalised self-care practices in daily live.

DISCUSSION

As illustrated in the findings, the personal, the situational, and the technical aspects of living with diabetes problematise the health care team's construction of participants being 'disengaged', ignoring that participants are constantly trying to balance diabetes and life priorities. This paper stresses the role health care teams can perhaps play to address social determinants and/or sociological problems along the life course, to promote CYP ability to build skills and competencies, whilst they search for a stable identity and seek intimacy outside family relationships.

At the centre of this discussion is the rebalancing of medicalised and non-medicalised self-care practices in CYP daily lives. The significance of this study is in arguing for a corrective emphasise, weighted evenly on the physiological and the psychosocial implications of diabetes. Medicalised and non-medicalised self-care practices are not diametrically opposed in the participants accounts, but instead negotiated and navigated on an evenly momentarily or periodic basis by CYP. What this means for health service design and delivery is clear, in that the NHS, rather than expecting CYP to adjust to health plans which are designed predominately by adults for CYP, health professionals should take into account the changing life circumstances and priorities of CYP in order to increase meaningful engagement in health services.

Recognition should also be paid to the reality that self-care extends far beyond what is discussed in hospital appointments or determined by reading of the CYP's blood sugar levels. The pressures of growing up in the twenty-first century weigh heavily on the self-

For peer review only - http://bmjopen.bmj.com/site/about/guidelines.xhtml

care strategies adopted by the participants, and in turn affect how well CYP adhere to medicalised self-care plans (e.g. doing carb counting, insulin injections, keeping to a healthy diet and regular exercise). The participant's accounts demonstrate how they are often preoccupied with the question of '*who I am'* and '*who I want to become'*. It goes without saying that the focus of most, if not all, of the participants has been on fitting in with their peer groups, thus often prioritising their social needs over their health needs.

Authors acknowledge that person-centred care planning is perhaps the right course forward; however, despite policy being in place in England, practice is ad hoc. Form this context, the challenge for health-care professionals is to create the right environment to design and deliver health-care plans that model effective shared decision making grounded in a person-centred approach that takes account of CYP life circumstances. Engendering trust in the clinical relationship ought to be prioritised to allow for honest and frank discussions on lifestyles, behaviour and identity, which are not currently actively taking place in clinical appointments.

The focus often remains on structured medical education and an expectation that overnight CYP become independent in their self-care. In practice, family and friends continue to play important roles in supporting CYP in managing their diabetes. Therefore, more emphasis should be placed on integrating both medicalised and non-medicalised self-care techniques in CYP personalised plans, with a specific focus on the functioning of the CYP support network.

Limitations

Only twenty-two out of forty-seven CYP identified by their clinicians as disengaged agreed to take part in the study. The size of the sample did not reflect the growing number of CYP with type 2 diabetes (Supplementary File), and perhaps a quota sampling strategy could have been use to ensure that the voices and issues for this discrete group were heard.

For peer review only - http://bmjopen.bmj.com/site/about/guidelines.xhtml

To conclude, this paper highlights that effective diabetes care in CYP requires more than a simple medical model. Diabetes teams need to appreciate the conflicting tensions experienced by CYP and to evolve better models for addressing their concerns regarding identity, risk and self-care in the context of their social setting and peer group. Exploring these issues and identifying ways to support CYP more effectively could reduce disengagement, improve health outcomes and make best use of healthcare resources.

For peer review only

Acknowledgements

The authors would like to thank all participants and healthcare providers who contributed their time to share their perspectives with us.

For peer review only - http://bmjopen.bmj.com/site/about/guidelines.xhtml

Contributors

All authors named contributed substantially to the document. DS and AH co-designed the study and interpreted the results. DS and MR wrote the draft manuscript. AH obtained the funding and provided support in editing the manuscript. DS, MR, AH, AM and VH contributed to the study design and critical review of the manuscript. All authors approved the final version.

For peer review only

Funding

This work was supported by the NHIR North Thames Collaborations for Leadership in Applied Health Research and Care

Competing interests

None declared.

Patient consent for publication

All the participants' (and parents) provided written consent or assent and all names have been pseudonymised.

Ethics approval

Ethical approval was obtained by NRES Committee South East Coast - Surrey Bristol Research Ethics Committee Centre (REC reference: 15/LO/0903 IRAS project ID: 179878)

Provenance and peer review

Not commissioned; externally peer reviewed.

For peer review only - http://bmjopen.bmj.com/site/about/guidelines.xhtml

PPI Statement

This study was co-designed and co-delivered with young people as peer researchers living with type 1 and 2 diabetes.

Data availability statement

N/A

REFERENCES

For peer review only - http://bmjopen.bmj.com/site/about/guidelines.xhtml

1. Toobert DJ, Hampson SE, Glasgow RE. The summary of diabetes self-care activities measure: results from 7 studies and a revised scale. *Diabetes Care* 2000;23(7):943-950. doi:10.2337/diacare.23.7.943

2. Alderson P, Sutcliffe K, Curtis K. Children as partners with adults in their medical care. *Arch Dis Child* 2006;91(4):300-303. doi:10.1136/adc.2005.079442

3. Fleming E, Carter B, Gillibrand W. The transition of adolescents with diabetes from the children's health care service into the adult health care service: a review of the literature. *J Clin Nurs* 2002;11(5):560-567. doi:10.1046/j.1365-2702.2002.00639.x

4. Curtis-Tyler K, Arai L, Stephenson T, Roberts H. What makes for a 'good' or 'bad' paediatric diabetes service from the viewpoint of children, young people, carers and clinicians? A synthesis of qualitative findings. *Arch Dis Child* 2015;100(9):826-833. doi:10.1136/archdischild-2014-307517

5. Kennedy I. Getting it right for children and young people: Overcoming cultural barriers in the NHS so as to meet their needs.2010. Available from: https://dera.ioe.ac.uk/881/1/dh_119446.pdf

6. Tabingo N, Verzone A. Improving compliance with diabetes care using a novel mnemonic: A quality improvement project in an urban primary care clinic. *Prim Care Diabetes* 2020;S1751-9918(20)30185-6. doi:10.1016/j.pcd.2020.04.008

7. Clark J, Nath V. Medical engagement: a journey not an event. King's Fund; 2014. Available from: https://www.kingsfund.org.uk/sites/default/files/field/field_publication_file/medical-engagement-a-journey-not-an-event-jul14_0.pdf

8. Spurgeon P, Clark J, Wathes R. Medical engagement and improving quality of care. *Future Hosp J* 2015;2(3):199-202. doi:10.7861/futurehosp.2-3-199

9. British Medical Association (BMA), Engagement within the NHS, London; 2017. Available from: https://www.bma.org.uk/media/2113/bma-engagement-within-the-nhs-brief-nov-2017.pdf

For peer review only - http://bmjopen.bmj.com/site/about/guidelines.xhtml

10. Elders V, Keen A, Gold A, et al. Adults with type 1 diabetes: what factors are associated with disengagement from health services?. *Pract Diab* 2014 Apr;31(3):117-8a. doi:10.1002/pdi.1845

11. Rhee MK, Slocum W, Ziemer DC, et al. Patient adherence improves glycemic control. *Diabetes Educ* 2005;31(2):240-250. doi:10.1177/0145721705274927

12. Masding MG, Klejdys S, MacHugh B, et al. Non-attendance at a diabetes transitional clinic and glycaemic control. *Pract Diab Int* 2010;27(3):109-10i. doi:10.1002/pdi.1456

13. Paterson BL, Charlton P, Richard S. Non-attendance in chronic disease clinics: a matter of non-compliance?. *J Nurs Healthc Chronic Illn* 2010;2(1):63-74. doi:10.1111/j.1752-9824.2010.01048.x

14. Jones SE, Hamilton S. The missing link: paediatric to adult transition in diabetes services. *Br J Nurs* 2008;17(13):842-847. doi:10.12968/bjon.2008.17.13.30535

15. Paterson BL, Russell C, Thorne S. Critical analysis of everyday self-care decision making in chronic illness. *J Adv Nurs* 2001;35(3):335-341. doi:10.1046/j.1365-2648.2001.01850.x

16. Gucciardi E. A systematic review of attrition from diabetes education services: strategies to improve attrition and retention research. *Can J Diabetes* 2008;32(1):53-65. doi:10.1016/S1499-2671(08)21011-7

17. Datye K, Bonnet K, Schlundt D, et al. Experiences of Adolescents and Emerging Adults Living With Type 1 Diabetes. *Diabetes Educ* 2019;45(2):194-202. doi:10.1177/0145721718825342

18. O'Brien BC, Harris IB, Beckman TJ, Reed DA, Cook DA. Standards for reporting qualitative research: a synthesis of recommendations. *Acad Med* 2014;89(9):1245-1251. doi:10.1097/ACM.0000000000000388

19. Robinson JA. Personal narratives reconsidered. *The journal of American folklore* 1981;94(371):58-85. doi:10.2307/540776

For peer review only - http://bmjopen.bmj.com/site/about/guidelines.xhtml

For peer review only

20. Deaux K, Reid A, Mizrahi K, et al. Parameters of social identity. *J Pers Soc Psychol* 1995;68(2):280.

21. Howard JA. Social psychology of identities. *Annu Rev Sociol* 2000;26(1):367-93. doi:10.1146/annurev.soc.26.1.367

22. Rose S, Paul C, Boyes A, et al. Stigma-related experiences in non-communicable respiratory diseases: A systematic review. *Chron Respir Dis* 2017;14(3):199-216. doi:10.1177/1479972316680847

23. Schabert J, Browne JL, Mosely K, et al. Social stigma in diabetes: a framework to understand a growing problem for an increasing epidemic. *Patient* 2013;6(1):1-10. doi:10.1007/s40271-012-0001-0

24. Smith LB, Terry A, Bollepalli S, et al. School-Based Management of Pediatric Type 1 Diabetes: Recommendations, Advances, and Gaps in Knowledge. *Curr Diab Rep* 2019;19(7):37. doi:10.1007/s11892-019-1158-x

25. Hinton D, Kirk S. Teachers' perspectives of supporting pupils with long-term health conditions in mainstream schools: a narrative review of the literature. *Health Soc Care Community* 2015;23(2):107-120. doi:10.1111/hsc.12104

26. Edwards D, Noyes J, Lowes L, et al. An ongoing struggle: a mixed-method systematic review of interventions, barriers and facilitators to achieving optimal self-care by children and young people with type 1 diabetes in educational settings. *BMC Pediatr* 2014;14:228. Published 2014 Sep 12. doi:10.1186/1471-2431-14-228

27. Hatherly K, Overland J, Smith L, et al. Providing optimal service delivery for children and adolescents with type 1 diabetes: a systematic review. *Pract Diab Int* 2009;26(4):154-9.

28. Kelo M, Martikainen M, Eriksson E. Self-care of school-age children with diabetes: an integrative review. *J Adv Nurs* 2011;67(10):2096-2108. doi:10.1111/j.1365-2648.2011.05682.x

For peer review only - http://bmjopen.bmj.com/site/about/guidelines.xhtml

29. Marks A, Wilson V, Crisp J. The management of type 1 diabetes in primary school: review of the literature. *Issues Compr Pediatr Nurs* 2013;36(1-2):98-119. doi:10.3109/01460862.2013.782079

30. Balfe M. The body projects of university students with type 1 diabetes. *Qual Health Res* 2009;19(1):128-139. doi:10.1177/1049732308328052

31. Sparapani Vde C, Borges AL, Dantas IR, et al. Children with Type 1 Diabetes Mellitus and their friends: the influence of this interaction in the management of the disease. *Rev Lat Am Enfermagem* 2012;20(1):117-125. doi:10.1590/s0104-11692012000100016

32. Banks GG, Berlin KS, Keenan ME, et al. How Peer Conflict Profiles and Socio-Demographic Factors Influence Type 1 Diabetes Adaptation. *J Pediatr Psychol* 2020;45(6):663-672. doi:10.1093/jpepsy/jsaa036

33. Salamon KS, Brouwer AM, Fox MM, et al. Experiencing type 2 diabetes mellitus: qualitative analysis of adolescents' concept of illness, adjustment, and motivation to engage in self-care behaviors. *Diabetes Educ* 2012;38(4):543-551. doi:10.1177/0145721712445214

34. Sherifali D, Viscardi V, Bai JW, et al. Evaluating the Effect of a Diabetes Health Coach in Individuals with Type 2 Diabetes. *Can J Diabetes* 2016;40(1):84-94. doi:10.1016/j.jcjd.2015.10.006

35. Tsiouli E, Alexopoulos EC, Stefanaki C, et al. Effects of diabetes-related family stress on glycemic control in young patients with type 1 diabetes: Systematic review. *Can Fam Physician* 2013;59(2):143-149.

36. Dashiff C, Morrison S, Rowe J. Fathers of children and adolescents with diabetes: what do we know?. *J Pediatr Nurs* 2008;23(2):101-119. doi:10.1016/j.pedn.2007.08.007

For peer review only

Reporting checklist for qualitative study.

Based on the SRQR guidelines.

Instructions to authors

Complete this checklist by entering the page numbers from your manuscript where readers will find each of the items listed below.

Your article may not currently address all the items on the checklist. Please modify your text to include the missing information. If you are certain that an item does not apply, please write "n/a" and provide a short explanation.

Upload your completed checklist as an extra file when you submit to a journal.

In your methods section, say that you used the SRQRreporting guidelines, and cite them as:

O'Brien BC, Harris IB, Beckman TJ, Reed DA, Cook DA. Standards for reporting qualitative research: a synthesis of recommendations. Acad Med. 2014;89(9):1245-1251.

		Reporting Item	Page Number
**Title**			1
	#1	Concise description of the nature and topic of the study identifying the study as qualitative or indicating the approach (e.g. ethnography, grounded theory) or data collection methods (e.g. interview, focus group) is recommended	
**Abstract**			2
	#2	Summary of the key elements of the study using the abstract format of the intended publication; typically includes background, purpose, methods, results and conclusions	
**Introduction**			4
Problem formulation	#3	Description and signifcance of the problem / phenomenon studied: review of relevant theory and empirical work; problem statement	

For peer review only

Purpose or research question	#4	Purpose of the study and specific objectives or questions	5/6
**Methods**			6
Qualitative approach and research paradigm	#5	Qualitative approach (e.g. ethnography, grounded theory, case study, phenomenolgy, narrative research) and guiding theory if appropriate; identifying the research paradigm (e.g. postpositivist, constructivist / interpretivist) is also recommended; rationale. The rationale should briefly discuss the justification for choosing that theory, approach, method or technique rather than other options available; the assumptions and limitations implicit in those choices and how those choices influence study conclusions and transferability. As appropriate the rationale for several items might be discussed together.	
Researcher characteristics and reflexivity	#6	Researchers' characteristics that may influence the research, including personal attributes, qualifications / experience, relationship with participants, assumptions and / or presuppositions; potential or actual interaction between researchers' characteristics and the research questions, approach, methods, results and / or transferability	6
Context	#7	Setting / site and salient contextual factors; rationale	6
Sampling strategy	#8	How and why research participants, documents, or events were selected; criteria for deciding when no further sampling was necessary (e.g. sampling saturation); rationale	6
Ethical issues pertaining to human subjects	#9	Documentation of approval by an appropriate ethics review board and participant consent, or explanation for lack thereof; other confidentiality and data security issues	7

For peer review only

Data collection methods	#10	Types of data collected; details of data collection procedures including (as appropriate) start and stop dates of data collection and analysis, iterative process, triangulation of sources / methods, and modification of procedures in response to evolving study findings; rationale	6
Data collection instruments and technologies	#11	Description of instruments (e.g. interview guides, questionnaires) and devices (e.g. audio recorders) used for data collection; if / how the instruments(s) changed over the course of the study	7
Units of study	#12	Number and relevant characteristics of participants, documents, or events included in the study; level of participation (could be reported in results)	Supplementary form
Data processing	#13	Methods for processing data prior to and during analysis, including transcription, data entry, data management and security, verification of data integrity, data coding, and anonymisation / deidentification of excerpts	7
Data analysis	#14	Process by which inferences, themes, etc. were identified and developed, including the researchers involved in data analysis; usually references a specific paradigm or approach; rationale	7
Techniques to enhance trustworthiness	#15	Techniques to enhance trustworthiness and credibility of data analysis (e.g. member checking, audit trail, triangulation); rationale	7
**Results/findings**			
Syntheses and interpretation	#16	Main findings (e.g. interpretations, inferences, and themes); might include development of a theory or model, or integration with prior research or theory	7
Links to empirical data	#17	Evidence (e.g. quotes, field notes, text excerpts, photographs) to substantiate analytic findings	7-14

For peer review only

Discussion

Intergration with prior work, implications, transferability and contribution(s) to the field	#18	Short summary of main findings; explanation of how findings and conclusions connect to, support, elaborate on, or challenge conclusions of earlier scholarship; discussion of scope of application / generalizability; identification of unique contributions(s) to scholarship in a discipline or field	15
Limitations	#19	Trustworthiness and limitations of findings	16

Other

Conflicts of interest	#20	Potential sources of influence of perceived influence on study conduct and conclusions; how these were managed	18
Funding	#21	Sources of funding and other support; role of funders in data collection, interpretation and reporting	18

None The SRQR checklist is distributed with permission of Wolters Kluwer © 2014 by the Association of American Medical Colleges. This checklist can be completed online using https://www.goodreports.org/, a tool made by the EQUATOR Network in collaboration with Penelope.ai

For peer review only

BMJ Open

Supporting disengaged children and young people living with diabetes to self-care: A qualitative study in a socially disadvantaged and ethnically diverse urban area

Journal:	*BMJ Open*
Manuscript ID	bmjopen-2020-046989.R1
Article Type:	Original research
Date Submitted by the Author:	01-Jul-2021
Complete List of Authors:	Sharpe, Darren; University of East London, Institute for Connected Communities (ICC)<br>Rajabi, Mohsen; University of Tehran<br>Harden, Angela; City University of London, Centre for Maternal and Child Health Research, School of Health Sciences<br>Moodambail, Abdul; Barts Health NHS Trust, Department of Paediatrics, Newham University Hospital<br>Hakeem, Vaseem ; Barnet and Chase Farm Hospitals NHS Trust, Royal Free London NHS Foundation Trust
**Primary Subject Heading**:	Public health
Secondary Subject Heading:	Public health
Keywords:	DIABETES & ENDOCRINOLOGY, Change management < HEALTH SERVICES ADMINISTRATION & MANAGEMENT, Community child health < PAEDIATRICS, Quality in health care < HEALTH SERVICES ADMINISTRATION & MANAGEMENT

SCHOLARONE™
Manuscripts

I, the Submitting Author has the right to grant and does grant on behalf of all authors of the Work (as defined in the below author licence), an exclusive licence and/or a non-exclusive licence for contributions from authors who are: i) UK Crown employees; ii) where BMJ has agreed a CC-BY licence shall apply, and/or iii) in accordance with the terms applicable for US Federal Government officers or employees acting as part of their official duties; on a worldwide, perpetual, irrevocable, royalty-free basis to BMJ Publishing Group Ltd ("BMJ") its licensees and where the relevant Journal is co-owned by BMJ to the co-owners of the Journal, to publish the Work in this journal and any other BMJ products and to exploit all rights, as set out in our licence.

The Submitting Author accepts and understands that any supply made under these terms is made by BMJ to the Submitting Author unless you are acting as an employee on behalf of your employer or a postgraduate student of an affiliated institution which is paying any applicable article publishing charge ("APC") for Open Access articles. Where the Submitting Author wishes to make the Work available on an Open Access basis (and intends to pay the relevant APC), the terms of reuse of such Open Access shall be governed by a Creative Commons licence – details of these licences and which Creative Commons licence will apply to this Work are set out in our licence referred to above.

Other than as permitted in any relevant BMJ Author's Self Archiving Policies, I confirm this Work has not been accepted for publication elsewhere, is not being considered for publication elsewhere and does not duplicate material already published. I confirm all authors consent to publication of this Work and authorise the granting of this licence.

For peer review only

Supporting disengaged children and young people living with diabetes to self-care: A qualitative study in a socially disadvantaged and ethnically diverse urban area

Darren Sharpe[1*], Mohsen Rajabi[1,2], Angela Harden[1,3], Abdul Moodambail[4], Vaseem Hakeem[5]

Institute for Connected Communities, University of East London, London, UK.
D.sharpe@uel.ac.uk

[2] Department of Psychology, University of Tehran, Tehran, Iran. Rajabimohsen@ut.ac.ir
ORCID: https://orcid.org/0000-0002-0254-9027

[3] School of Health Sciences, City, University of London, London, UK.
angela.harden@city.ac.uk ORCID: 0000-0002-8621-5066

[4] Consultant Pediatrician, Evelina London Children's Hospital, Guy's and St Thomas' Foundation Trust, London. UK. abdulr@doctors.org.uk

[5] Consultant Pediatrician , Barnet Hospital, Royal Free London NHS Foundation Trust, London, UK. Vaseem.hakeem@nhs.net

*Corresponding Author: Institute for Connected Communities, University of East London, Stratford Campus, Water Lane, London, E15 4LZ, UK.
Email address: D.sharpe@uel.ac.uk
ORCID: https://orcid.org/0000-0001-7418-4496
Telephone: +442082234325

Word Count:

For peer review only - http://bmjopen.bmj.com/site/about/guidelines.xhtml

5,337

Abstract

Objective: To explore how to enhance services to support the self-care of children and young people (CYP) clinically considered 'disengaged' by diabetes services.

Design: Qualitative study

Setting: Two diabetes clinics in an ethnically diverse and socially disadvantaged urban area in the UK. Eligible participants were CYP living with Type 1 or Type 2 diabetes aged between 10 and 25 years who did not attend their last annual hospital appointment.

Participants: 22 CYP (14 female and 8 male) aged between 10 and 19 years old took part. The sample was diverse in terms of ethnicity, age at diagnosis, family composition, and presence of diabetes amongst other family members.

Data collection: Semi-structured interviews

Data analysis: Data were analysed thematically.

Results. Analysis of participant accounts confirmed the crucial importance of non-medicalised care in children and young people diabetes care. A life plan was considered as important to participants as a health plan. Participants valued the holistic support provided by friends, family members and school teachers. However, they found structural barriers in their health and educational pathways as well as disparities in the quality of support at critical moments along the life course. They actively tried to maximise their well-being by balancing life priorities against diabetes priorities. Combined, these features could undermine participants engagement with health services where personal strategies were often held back or edited out of clinical appointments in fear of condemnation.

Conclusion. We demonstrate why diabetes health teams need to appreciate the conflicting pressures experienced by CYP and to co-produce more nuanced health plans for addressing their concerns regarding identity and risk taking behaviours in the context of their life-worlds. Exploring these issues and identifying ways to better support CYP to address them more pro-actively should reduce disengagement and set realistic health outcomes that make best use of medical resources.

For peer review only - http://bmjopen.bmj.com/site/about/guidelines.xhtml

Keywords:

Disengagement, children and young people, diabetes services, self-care

Strengths and limitations of this study

- CYP who are usually marginalised and rarely heard in research were successfully recruited in this study including those from minority ethnic groups and those identified as ‘disengaged’ from health services.
- Diversity could have been increased further through recruitment of greater numbers of CYP with Type 2 diabetes.
- The in-depth interviews were scheduled at times and locations chosen by the young person which helped to avoid replicating the power dynamics experienced in clinical appointments.
- The in-depth interview format was co-designed with a PPI group, which supported CYP to talk openly on matters that concerned them most about diabetes self-care.
- The thematic framework was informed by both previous research *and* inquiry workshops with CYP led by the PPI group.

For peer review only - http://bmjopen.bmj.com/site/about/guidelines.xhtml

INTRODUCTION

"Appointments can feel a bit intimidating – especially when there are five or six people in the room at the time. I do not like feeling judged or stared at. We should be able to have an open discussion about diabetes in relation to my risk-taking behaviour."

(Participant 17, aged 13)

Diabetes self-management in children and young people (CYP) is a concern because of the assumption that establishing a healthy lifestyle will increase better metabolic control of diabetes and will reduce the risk of complications in adulthood. Diabetes self-care includes a range of activities (e.g., self-monitoring of blood glucose, eating a low-saturated-fat diet, and checking one's feet), and it is now well established that these different components do not correlate highly [1]. In spite of structured education programmes and regular health promotion messages made by health professionals, researchers and charities, we seldom hear the voices of CYP living with diabetes. We do not hear how they approach food and exercise alongside navigating the physiological and psychological changes consistent with growing up, or how they get their voices heard within health services in which we see a culture that is arguably dominated by paternalism [2–5]. The experience of being 'silenced' can often be compounded by parents, who do not give power away for putting their son's/daughter's health care plan into practice. Thus, for CYP living with diabetes, taking ownership of their self-care is far more complex than adherence to a health plan; they must negotiate and navigate a number of different relationships and contexts.

Research highlights the negative long-term health outcomes for members of the public who disengage from health services [6–8]. According to the UK National Health Service (NHS) 'disengagement' is defined when a CYP, or carer does not respond to requests from health professionals [9]. Behaviours of disengagement are usually cumulative and

For peer review only - http://bmjopen.bmj.com/site/about/guidelines.xhtml

may include: disregarding health appointments; not having a GP; not being home for professional visits; not allowing professionals into the home; agreeing to take action but never doing it; hostile behaviour towards professionals; manipulative behaviour resulting in no health care; actively avoiding contact with professionals; and attendance at urgent care centres, accident and emergency departments but not waiting to be seen/taking own discharge [9]. Elders [10] argues that people most likely to disengage are characteristically, "young; are from more deprived areas; are more commonly anxious and depressed; have higher HbA1c values; and are more frequently male" (p115).

Much of the medical literature on non-attendance in diabetes points to significantly higher HbA1c results amongst so-called 'defaulters' used as an example of the benefits of clinic attendance [11]. In England, there is evidence that young people miss more scheduled medical appointments of all kinds than other age groups [12,13]. Indeed, for younger patients, the transition from paediatric to adult diabetes clinics, is highlighted as a critical moment when young people drop out of the system [14]. However, reviews of the existing literature show a limited number of studies that have assessed the reasons behind clinic non - attendance, from the young patients' perspectives [15,16]. Understanding the challenges of CYP living with diabetes, from their perspective, especially in areas where a disproportionate burden of cases in diabetes falls on those from ethnic minority groups is needed. Most of the existing research does not focus on diverse groups of CYP or those from socially disadvantaged groups. So, this is about learning from those marginalised and/or not seen or heard voices in existing research [15-17]. This is an important first step to help shape the right diabetes care, at the right time, for CYP who live in communities that experience high deprivation and health inequalities.

This study was undertaken in response to a whole system call to improve accessible care for CYP with Type 1 or Type 2 diabetes living in London by understanding the barriers and identifying solutions to increase self-management. We focused on two boroughs within

For peer review only - http://bmjopen.bmj.com/site/about/guidelines.xhtml

London, one of which has the highest local prevalence of type 2 diabetes in those between 16 and 25 years in the UK [18].

Through qualitative methods this study seeks to deepen understanding into the barriers and drivers behind disengagement from services for CYP from socially deprived and ethnically diverse communities since these populations are most at risk of experiencing disparities in health provision and outcomes resulting from structural barriers. We examine how these barriers can be overcome and analyse CYPs independent self-care practices to explore what diabetes services can do to optimise safe self-care amongst this group.

METHODS

Design

In-depth interviews with CYP with Type 1 or Type 2 diabetes were used to assess their understanding and experiences of attending clinics and living with diabetes. The study was part of a larger programme of work undertaken between May 2016 to May 2018 focused on co-designing diabetes services for children and young people. This broader programme included a systematic review of reviews, inquiry workshops and the development and evaluation of a young commissioner model in which young people with diabetes worked alongside adults to commission diabetes services [19]. The study followed steps recommended by the SRQR guideline [20].

Patient and Public Involvement

The study was led by a team of adult researchers with the help of a group of young co-investigators aged between 16 and 25 living with Type 1 or 2 diabetes. The young co-investigators reviewed all study documentation including the study protocol, participant information sheets and the interview topic guide but they were not involved in the recruitment to and conduct of the study. Some members of this group also took on young commissioner roles within the larger programme in which this qualitative study is nested. The young co-investigators met regularly over the study period (at least monthly) and

For peer review only - http://bmjopen.bmj.com/site/about/guidelines.xhtml

received training in Diabetes 101, public speaking, workshop facilitation and commissioning public services. Findings of the overall programme were fed back to study participants via research briefings, a webinar series and public engagement events with targeted schools and diabetes networks attended by study participants and health professionals.

Sampling and recruitment

We used a non-probability sampling strategy to identify, from two diabetes clinic registers, CYP categorised as 'disengaged' by their clinician as per the NHS Policy of Engagement and Disengagement with services definition [9]. Potential participants were first approached by a member of the health care team, followed by a screening call from the research team. If the CYP were eligible (i.e. they were aged between 10 and 25; living with Type 1 or Type 2 diabetes; living in North or East London; were pre or post a relevant transition such as moving from primary to secondary schools or moving to adult services; and did not attend their last annual hospital appointment) they were invited to take part in an interview.

A total of 22 CYP aged 10 to 19 years took part in the study out of 47 approached by the research team. Just over half of the sample were female and there was diversity in terms of age, ethnicity, age at diagnosis with diabetes, family composition, and presence of diabetes amongst other family members (Table 1). All but two of the participants were living with Type 1 diabetes.

Table 1: Characteristics of interview participants

	N		N
**Gender**		**Number of siblings**	
Male	8	0	3

For peer review only - http://bmjopen.bmj.com/site/about/guidelines.xhtml

Female	14	1	12
**Age**		2	3
10-13	9	3	2
14-17	4	>3	2
18 and over	9	**Position in family**	
**Ethnicity**		Eldest	8
Black African	2	Second Eldest	4
Black Caribbean	2	Second youngest	1
Dual Heritage	4	Youngest	6
South Asian	7	Only child	3
White British	6	**Adults in the household**	
White Other	1	Mother only	4
**Diabetes type**		Mother and father	15
Type 1	20	Other	3
Type 2	2		
**Age at diagnosis**		**Other family members with diabetes**	
0 to 4	8	None	12
5 to 9	5	One other	4
10 to 15	9	More than one	6

For peer review only

Data collection

Semi-structured interviews were conducted to explore the personal journeys and lifestyles of CYP living with diabetes using a topic guide. Participants were asked:

- to describe their journey from diagnosis to now, including how they got diagnosed, how they felt, and what they would like to be different;

For peer review only - http://bmjopen.bmj.com/site/about/guidelines.xhtml

- what worked really well and what made life harder for participants that could be changed;
- how they managed their condition and what the health service (GPs, nurses, and doctors) and other organisations (schools, youth centres, and sports clubs) could do to more or less to positively impact on their life; and
- their future vision for diabetes services for children and young people.

The majority of interviews took place in participant's homes (N=15) with five taking place at the university campus, one over the telephone and one at the participant's school. In half of the interviews (N=11) another family member was also present, most frequently the mother (N=7). Interviews were digitally recorded and transcribed.

Data analysis

We analysed interview data using thematic analysis to find patterns of meaning that helped to explain CYPs 'disengagement' covering three focal points [21] The three areas included their diabetes biography, social networks and future hopes and aspirations. Analysis involved repeated readings of the transcripts to gain familiarity with the content, the use of coding to identify recurring, similar and contrasting content, and the collapsing of codes into central themes by three members of the research team. Data validation was achieved by double coding a proportion of the transcripts followed by bringing all the coded transcripts into calibration meetings where we discussed and debated the constitution of each emergent theme and sub-theme. Our coding scheme was also informed by insights from the systematic review of reviews and inquiry workshops with children and young people led by our young co-investigators from the wider programme of work and from health professionals and commissioners' insights from practice who formed part of the project task group.

Ethics

For peer review only - http://bmjopen.bmj.com/site/about/guidelines.xhtml

Ethical approval was granted by NRES Committee South East Coast-Surrey (15/LO/0903) in combination with the Health Research Authority (179878). Informed written consent was sought and received from all participants. A participant information sheet and consent form were sent to all potential participants at least 48 hours before a scheduled interview to allow time for them to consider their participation and ask any questions. On the day of the interview participants were given a further opportunity to ask questions before signing a consent form. For potential participants aged 10 to 15 signed consent/permission was sought from their parent/legal guardian for their son/daughter to be approached to take part in the study. These younger participants were given information about the research project and were invited to sign an assent form following permission from their parent/carer. Findings and quotes in the report are pseudo-anonymised to minimise the risk of identifying participants.

Results

This section illustrates where and how participants negotiate and navigate the pressures from health services, education, home and social networks as part of their diabetes self-care. To recap, forty-seven potential interviewees agreed with their adult gatekeeper to be contacted by the research team out of which twenty-two were successfully interviewed. Potential interviewees dropped out of the study due to episodes of homelessness, changing school diaries, and failure to show up to agreed interview appointments. The interviewed participants identified a range of issues they faced in living with diabetes including balancing the management of their diabetes with other aspects of their lives (e.g. school, college or university, sports and hobbies), problems with maintaining glycemic control (e.g. difficulties carb counting, not liking healthy foods) and problems encountered within diabetes clinics (e.g. poor relationships with clinic staff). The following themes were identified from participant interviews.

Stigmatized status

For peer review only - http://bmjopen.bmj.com/site/about/guidelines.xhtml

Participants' shared common stories of how their peer groups behave, look and think. Accounts of peer group norms revealed how participants positioned themselves at the centre and/or margins of such norms rather than the actual perceptions and behaviour of their peers. Participants said:

> If I was to walk on the street, no one could actually tell that I had diabetes, unless I actually do something … for example a blood test… (P7, aged 15, T1).

> Now at age 15, I have realised that diabetes doesn't make me different from anybody else (P10, aged 15, T1).

The relationship with self and others is complex and can positively and negatively impact on self-care. One participant explains:

> I was sixteen and, on the bus, and I just didn't feel well, and I did my injection and the woman [passenger] said, "oh my god", and I was, like, sorry yeah? And they're like, "you know it's not good to take drugs on the bus?" And I'm like, "excuse me", and my friend just started laughing like. Cos we couldn't believe it and I'm like "it's not drugs. I'm diabetic" (P16, aged 17, T1).

Being a young person with diabetes was not the only marker of difference. Participants identified as belonging to ethnic minority groups (or linguistic groups), overlaid with intra group differences based on their gender, as well as lifestyle differences bought upon by living with diabetes. Our analysis has shown that the perception of social stigma (e.g. disapproval of a person based on perceived social characteristics) is a common feature negotiated in how participants experience building a stable social identity [22,23] whilst managing a chronic disease [24,25].

For peer review only - http://bmjopen.bmj.com/site/about/guidelines.xhtml

For street-involved participants, the need to not appear vulnerable and to belong is often the reason given to why they might deviate from their health plans in order to navigate territorial stigmatisation of their identity and to increase the likelihood of inclusion by peers and in wider society. Ironically, participants reported feeling a greater sense of wellbeing, feeling safe and feeling valued in ethnically and/or religiously defined spaces than inside the clinic room, where they felt scrutinised and compartmentalised.

Faith-based identity

Participants who identified as Christian or Muslim reported that their belief system played a significant role in their self-care, which could have both positive and negative consequences for the management of their diabetes. For example, participants gave accounts where religious observance helped them with their self-care. One participant said:

> As a Muslim you can't drink, [and I] don't smoke anyway, but there are rules like that, and, like, I do go out with friends but it's [drinking and smoking], not something I majorly think about (P13, aged 18, T1).

In contrast, two participants said:

> [Ramadan] is a sacred month for us. So, yeah, I want to be part of that sacred thing as well, so that's why I feel upset when I can't do it as well (P7, aged 15, T1).

> I had a DKA [diabetic ketoacidosis] over Ramadan. My mother was away and I was at home with my older sister. I wanted to experience fasting and the feast at the end. However, I ended up in hospital (P11, aged 18, T2).

Central to most, but not all, the participants' accounts was the importance they attached to their faith-based identities and how normative practices shared across their faith

For peer review only - http://bmjopen.bmj.com/site/about/guidelines.xhtml

communities have been performed and, on occasion, have resulted in positive and negative self-care. Ramadan is an exemplifier of one such practice that can serve to resource identity but can also undermine healthy behaviours, so too are many other festivities and cultural events that require a break from routine eating patterns. Clinical teams should acknowledge the cultural spaces in which CYP inhabit and expect deviations from idealized medical plans.

Supporting a CYP with diabetes in education

In the participants accounts of performing self-care their educational experiences were central features, with a specific focus on how teachers act as proxy health care workers. Participants said;

> …my tutor helps me a lot cos I have to test at the right time, and sometimes I forget, so my tutor is there to help me if I'm doing it right or I'm doing it wrong (P3, aged 11, T2).

> At school I use, like, a special room. I would disappear into it [to do injections].
> (P9, aged 15, T1)

> They [school] have an insulin register. If anybody is missing on the register, they go and look for them and remind them, have you eaten? (P7, aged 15).

Participants highlighted how policies and procedures in schools in poor communities, have helped to support them in their self-care. However, participants' accounts show challenges to self-care when transitioning between schools and then onto college and University. Participants mentioned:

> Going from primary to secondary school and then college is difficult in relation to the information and knowledge they [educators] have about my diabetes. …you receive less

For peer review only - http://bmjopen.bmj.com/site/about/guidelines.xhtml

and less help and therefore educators know less and less about diabetes. (P11, aged 18, T2)

I experienced problems moving away from home to university and trying to sort out my studies, prescriptions and doctor appointments. (P2, aged 18, T1)

Participants highlighted the positive and negative features of school-based diabetes support [26], with teachers playing a crucial role [27]. However, participants also observed a lack of training opportunities in schools to help teachers to improve their knowledge of diabetes care [28–31]. The overly reported challenge has been in transitions, when ironically support tends to tail off [32]. What is missing is a coordinated approach led by the health team in preventing the marginalisation of the CYPs health plans especially during stressful life events in their educational journeys, which result in high or imbalanced sugar levels leading to hospitalisation. In practice, participants have tended to figure things out for themselves when the health care team could help in a proactive way to produce a smooth transition.

Friendship networks, and not the health care team - formed a conceptual bridge between home and school, which was felt to be especially important to CYP with diabetes [33,34]. Peers are a central factor in a child's socialization whether they have diabetes or not, but there are few studies addressing the role of friends among CYP in the management of the disease. A selection of accounts illustrates the type of support provided by friends in the self-care of participants in this study. Participants said:

I collapsed in the middle of the playground and he [best friend] was taught by my mum what to do. So, then he called my mum, who told him to call 999. (P7, aged 15, T1)

…when I know I'm going out for a drink with friends, I make sure that I have my meal and my sugar levels are good. When you're drinking, obviously I don't get in to a state where I

For peer review only - http://bmjopen.bmj.com/site/about/guidelines.xhtml

don't know what I'm doing like. I'm scared to be, like, overly drunk and waking up in a diabetic coma or something. (P19, aged 18, T1)

Participants' accounts reveal how they use friendship networks as a source of support in their self-care, as argued by Salamon [35]. Yet still, social isolation and loneliness effects CYP and a few of the participant's experienced weak friendship networks and did not know other CYP in their cohort living with diabetes to relate too. Participants remark:

I think as a child you want to really open up and talk to someone that understands what you're going through, understands the injections, and the needles. I think when I first got diabetes, I didn't know anyone with it so your kind of like, you can't talk about it with anybody. (P16, aged 17, T1)

I guess I don't get any real support. I have a couple of friends on Facebook, and we basically help each other through anything. (P10, aged 15, T1)

On the surface, health care teams appear absent in helping to educate friends or lack knowledge of the support provided by friends for CYP in their care. The home environment was important to understanding how participants made decisions affecting their self-care. The study takes as given the emotional consequences for CYP living in families undergoing sustained economic strain. CYP living in poverty are more likely to feel like a failure and have a sense of hopeless about their future than their more affluent peers. As a result, participants' choices around their self-care have often been made against the backdrop of health inequalities. In the face of economic hardships participants provided rich descriptions of how family members often serve as a crucial source of support in their self-care. Participants said;

I have lived with diabetes for the last thirteen years. It hasn't always been under control. It was when my mother took responsibility for me that my diabetes was more controlled, more freedom means less control. (P11, aged 18, T2)

For peer review only - http://bmjopen.bmj.com/site/about/guidelines.xhtml

> I feel like because they [older family members] were like born with it [diabetes], they don't really talk to us about it, you know, how it's affected them or what they do. (P4, aged 12, T1)

> I go to the gym with my auntie. It's like a peace of mind away from home. They have the little TV screens in front of each activity and on the cross trainer and the treadmill, and on the steps and things. So, I can just watch TV (P8, aged 18).

Seldom do studies capture the schooling experience in poor communities and how family circumstances reduce parental ability for active involvement in their child's self-care in school, especially from ethnic minority families. Evidence suggests that conscientiously caring for the carer helps CYP to reach their full potential in increasing resilience living with diabetes. This is reflected in: psychological models of assessment of care givers; understanding illness belief systems [36]; interdisciplinary working to help reduce family stress and anxiety [37]; including fathering a child with diabetes [38]. The increasing diversity experienced in urban areas such as London necessitates the cultural competency of the health care workers into the needs of families living in economic stress in order to provide the right support at the right time to care givers.

Diabetes self-management

Too often, participants behaviour and attitudes have been wrongly characterised by clinicians as a marker of their disengagement from the service. The markers are hardly ever directly discussed by the health care team or voluntarily disclosed in clinical appointments by CYP but loom large over the relationship. Participants said:

> At first, I didn't really take it [diabetes] that seriously, I suppose, but then when you realise it's going to affect you every day, then I started to take it more seriously. (P9, aged 15, T1)

For peer review only - http://bmjopen.bmj.com/site/about/guidelines.xhtml

> I try not to eat as much, so that my blood sugar level doesn't go so high and [it] saves me from having [to] reinject myself with more insulin. (P6, aged 13, T1)

> I'll eat what I want, I'll drink what I want and that's when my sugar levels started running high and I was just uncontrolled. So, I was missing appointments sometimes, like, oh, I'm just going to miss, I don't want to go. (P2, aged 18, T1)

> There are certain things you must cut back on. I used to do boxing, which was intense training, for an hour or so, so I think stuff like that I kind of left. I haven't been doing it as much. I feel it's quite a lot harder to do with diabetes. (P16, aged 17, T1)

In the latter case, no clinical support was provided to the participant to learn how to cope with high intensity exercise, in order to relieve the anger that so many of participants felt living with the condition. We see missed opportunity by health care teams to educate and support CYP to build liveable lives. Also, participants, highlight the inherent complications in implementing their health plan. Participants said;

> I'm constantly having to remember to do my blood test, and do my injections. Plus, with me, when I do my injection, after doing it, it does hurt. I get a bit frustrated and you don't want to do it afterwards. (P4, aged 12, T1)

> Most of the time, I find [doing injections] easy. Sometimes it's kind of annoys me that I must keep doing it and doing it, and sometimes it hurts, and, yeah, fear and perceptions of doing injections. (P2, aged 18, T1)

> You can't really tell I'm wearing a pump right now because everything is electronic blue tooth? I know that the pump and the meter will connect, automatically vibrate, so I will know the insulin will get delivered. Whereas, with the pen, I have to pick up my clothes, to open this, put a needle in the pen, do this, it's like a big long procedure before I actually give myself the injection. (P7, aged 15, T1)

For peer review only - http://bmjopen.bmj.com/site/about/guidelines.xhtml

Using technology in self-care is proposed to CYP as a way to promote better self-care; however, this benefit is not borne out in this study. Indeed, Balfe [29] highlights how little attention has been paid to the accounts of CYP of the reasons why they may experience difficulties with their diabetes technology. Participants said:

> I tried it [pump] for a while, but it's very painful. It can be an inconvenience practically. I was doing PE, for example, and I had it on, it would just be annoying because I couldn't play football or anything. (P20, aged 18, T1)

> I'm getting use to putting my carb count in the machine so it does the maths for me. I'm not really good at that because I like doing it mentally and, like, turn off the machine straight away. (P7, aged 15, T1)

The benefits of technology (e.g. e-health) to aid self-care are inconclusive. Technological determinism is both embraced and resisted in the participants self-care narratives of Type 1 participants. More examination needs to be undertaken exploring how today's CYP from poor and ethnically diverse communities navigate self-care, identity, and intimacy in a digital world.

The five principle circumstances or markers that, so far as we have been able to observe, that drive the extent to which CYP with Type , and a lesser degree Type 2, are able to engage with health services and self-care include: (1) the probability or improbability of achieving normalcy when confronted with social stigma; (2) the easiness or difficulty in integrating faith-based practices into their daily routines; (3) the consistency or inconsistency in the quality of support provided in education; (4) the constancy or inconstancy of family and friends; and (5) the obstacles and practical challenges in balancing medicalised self-care practices in daily live.

For peer review only - http://bmjopen.bmj.com/site/about/guidelines.xhtml

DISCUSSION

This study has systematically collected and considered CYPs perspective on diabetes services and factors influencing their self-care. Listening carefully to CYP, who had all been categorised as becoming disengaged with their diabetes care teams, has enabled the research team to amplify their voices and, through the wider programme of work in which this qualitative study is nested, informed commissioning guidance to improve diabetes services for CYP living in a poor and ethically dense communities in London. It is well-recognised that CYP with long-term conditions may disengage from clinical services during adolescence and emerging adulthood. For CYP this can often contribute to poor health outcomes and for services a waste of medical resources.

Psycho-social needs and priorities of CYPs with Type 1 diabetes and barriers to their engagement in educational settlings [39] have been widely reviewed in the literature [40, 41]. Most of these factors are centred around CYPs desire to lead a 'normal' life like their peers and must awkwardly adjust their language and behaviour to be considered as 'normal and healthy'. These factors may be less salient for those with Type 2 diabetes as the management of their condition does not usually involve injections or pumps. Similarly, variations of the dynamics of engagement in self-care among Black and Ethnic minority groups have been discussed in the literature that emphasis peer pressure to meet sub-cultural expectations, which is shown to undermine health plans, and reinforce a lack of trust health professional [42].

In contrast, the recent Commission for Race and Ethnicity Report in the UK [43,44] noted that the majority of all ethnic groups – which leave out the voices of CYP- reported positive experiences of access to healthcare and concludes that ethnic minority groups have better outcomes than the white population despite experiencing higher levels of deprivation. The factors at play are complex, and one way to account for the positive stories of self-care reported through this study - despite broken engagement with clinics - is through the

For peer review only - http://bmjopen.bmj.com/site/about/guidelines.xhtml

cultural competencies narrated by participants in this study that have been applied to their self-care. For instance, Islamic law doesn't strictly forbid smoking, but it clearly stated that smoking is not good for health, and the health benefits of the Caribbean diet.

As illustrated in the findings, the personal, the situational, and the technical aspects of living with Type 1 and Type 2 diabetes problematise the simplistic and often unusual label given to Black, Asian and minority ethnic group CYP who are considered to be 'disengaged', ignoring that participants are constantly trying to balance diabetes and life priorities often as a visible 'Other' [45]. This paper stresses the role health care teams can perhaps play to address social determinants and/or sociological problems along the life course, to recognise CYP strengths and competencies, whilst they search for a stable identity and seek intimacy outside family relationships [46].

At the centre of this discussion is the rebalancing of medicalised and non-medicalised self-care practices in CYP daily lives. The significance of this study is in arguing for a corrective emphasise, weighted evenly on the physiological and the psychosocial implications of diabetes for CYP from deprived and ethically diverse communities. Medicalised and non-medicalised self-care practices are not diametrically opposed in the participants accounts, but instead negotiated and navigated on an evenly momentarily or periodic basis. What this means for health service design and delivery is clear; rather than expecting CYP to adjust to health plans which are designed predominately by adults for CYP, health professionals should take into account the changing life circumstances and cultural priorities of CYP in order to increase meaningful engagement in health services.

Recognition should also be paid to the reality that self-care extends far beyond what is discussed in hospital appointments or determined by reading of CYPs blood sugar levels. The pressures of growing up in the twenty-first century weigh heavily on the self-care strategies adopted by the participants, and in turn affect how well CYP adhere to

For peer review only - http://bmjopen.bmj.com/site/about/guidelines.xhtml

medicalised self-care plans (e.g. doing carb counting, insulin injections, keeping to a healthy diet and regular exercise). The participant's accounts demonstrate how they are often preoccupied with the question of '*who I am'* and '*who I want to become'*. It goes without saying that the focus of most, if not all, of the participants has been on fitting in with their peer groups, thus often prioritising their social needs over their health needs.

Person-centred care planning is a promising way forward; however, despite policy being in place in England, practice is ad hoc. Form this context, the challenge for healthcare professionals is to create the right environment to design and deliver health-care plans that model effective shared decision making grounded in a person-centred approach that takes account of CYP life circumstances. Engendering trust in the clinical relationship ought to be prioritised to allow for honest and frank discussions on lifestyles, behaviour and identity, which are not currently actively taking place in clinical appointments. Thus, more emphasis should be placed on integrating both medicalised and non-medicalised self-care techniques in CYP personalised plans, with a specific focus on the functioning of the CYP support network.

To conclude, this paper argues for non-medicalised self-care to gain parity with medicalised forms. In other words, a life plan is considered as important to the CYP as a health plan. We explore how both medicalised and non-medicalised forms of self-care often intersect in the daily lives of ethnically diverse CYP living in disadvantaged areas, and what this means for health care professionals. Diabetes teams need to appreciate the conflicting tensions experienced by CYP and to evolve better models for addressing their concerns regarding identity, risk and self-care in the context of their social setting and peer group. Exploring these issues and identifying ways to support CYP more effectively could reduce disengagement, improve health outcomes and make best use of healthcare resources.

For peer review only - http://bmjopen.bmj.com/site/about/guidelines.xhtml

For peer review only

Acknowledgements

The authors would like to thank all participants and healthcare providers who contributed their time to share their perspectives with us. We would like to thank members of the research team Dr Emma Green and James Rudd. Most importantly we would like to thank our team of young co-investigators Sema Thasnim, Shandies Rose, Tahmid Alam and Khadija Miah.

Contributors

All authors named contributed substantially to the document. DS and AH co-designed the study. DS and EG collected the data and DS, AH, EG and JR analysed the data. DS and MR wrote the draft manuscript. AH obtained the funding and provided support in editing the manuscript. DS, MR, AH, AM and VH contributed to the design and critical review of the manuscript. All authors approved the final version.

Funding

This work was supported by the NHIR North Thames Collaborations for Leadership in Applied Health Research and Care. The views expressed are those of the author(s) and not necessarily those of the NHS, the NIHR or the Department of Health and Social Care.

Competing interests

None declared.

Patient consent for publication

All the participants' (and parents) provided written consent or assent and all names have been pseudonymised.

For peer review only - http://bmjopen.bmj.com/site/about/guidelines.xhtml

Ethics approval

Ethical approval was obtained by NRES Committee South East Coast - Surrey Bristol Research Ethics Committee Centre (REC reference: 15/LO/0903 IRAS project ID: 179878)

Provenance and peer review

Not commissioned; externally peer reviewed.

PPI Statement

This study was co-designed and co-delivered with young people as peer researchers living with Type 1 and 2 diabetes.

Data availability statement

The interview audios and transcripts are not available to share outside of the research team.

For peer review only - http://bmjopen.bmj.com/site/about/guidelines.xhtml

REFERENCES

1. Toobert DJ, Hampson SE, Glasgow RE. The summary of diabetes self-care activities measure: results from 7 studies and a revised scale. *Diabetes Care* 2000;23(7):943-950. doi:10.2337/diacare.23.7.943

2. Alderson P, Sutcliffe K, Curtis K. Children as partners with adults in their medical care. *Arch Dis Child* 2006;91(4):300-303. doi:10.1136/adc.2005.079442

3. Fleming E, Carter B, Gillibrand W. The transition of adolescents with diabetes from the children's health care service into the adult health care service: a review of the literature. *J Clin Nurs* 2002;11(5):560-567. doi:10.1046/j.1365-2702.2002.00639.x

4. Curtis-Tyler K, Arai L, Stephenson T, Roberts H. What makes for a 'good' or 'bad' paediatric diabetes service from the viewpoint of children, young people, carers and clinicians? A synthesis of qualitative findings. *Arch Dis Child* 2015;100(9):826-833. doi:10.1136/archdischild-2014-307517

5. Kennedy I. Getting it right for children and young people: Overcoming cultural barriers in the NHS so as to meet their needs.2010. Available from: https://dera.ioe.ac.uk/881/1/dh_119446.pdf

6. Tabingo N, Verzone A. Improving compliance with diabetes care using a novel mnemonic: A quality improvement project in an urban primary care clinic. *Prim Care Diabetes* 2020;S1751-9918(20)30185-6. doi:10.1016/j.pcd.2020.04.008

7. Clark J, Nath V. Medical engagement: a journey not an event. King's Fund; 2014. Available from: https://www.kingsfund.org.uk/sites/default/files/field/field_publication_file/medical-engagement-a-journey-not-an-event-jul14_0.pdf

8. Spurgeon P, Clark J, Wathes R. Medical engagement and improving quality of care. *Future Hosp J* 2015;2(3):199-202. doi:10.7861/futurehosp.2-3-199

For peer review only - http://bmjopen.bmj.com/site/about/guidelines.xhtml

9. British Medical Association (BMA), Engagement within the NHS, London; 2017. Available from: https://www.bma.org.uk/media/2113/bma-engagement-within-the-nhs-brief-nov-2017.pdf

10. Elders V, Keen A, Gold A, et al. Adults with type 1 diabetes: what factors are associated with disengagement from health services?. *Pract Diab* 2014 Apr;31(3):117-8a. doi:10.1002/pdi.1845

11. Rhee MK, Slocum W, Ziemer DC, et al. Patient adherence improves glycemic control. *Diabetes Educ* 2005;31(2):240-250. doi:10.1177/0145721705274927

12. Masding MG, Klejdys S, MacHugh B, et al. Non-attendance at a diabetes transitional clinic and glycaemic control. *Pract Diab Int* 2010;27(3):109-10i. doi:10.1002/pdi.1456

13. Paterson BL, Charlton P, Richard S. Non-attendance in chronic disease clinics: a matter of non-compliance?. *J Nurs Healthc Chronic Illn* 2010;2(1):63-74. doi:10.1111/j.1752-9824.2010.01048.x

14. Jones SE, Hamilton S. The missing link: paediatric to adult transition in diabetes services. *Br J Nurs* 2008;17(13):842-847. doi:10.12968/bjon.2008.17.13.30535

15. Hilliard ME, Perlus JG, Clark LM, Haynie DL, Plotnick LP, Guttmann-Bauman I, Iannotti RJ. Perspectives from before and after the pediatric to adult care transition: a mixed-methods study in type 1 diabetes. *Diabetes Care* 2014;37(2):346-54. doi: 10.2337/dc13-1346.

16. Simms M, Baumann K, Monaghan M. Health Communication Experiences of Emerging Adults with Type 1 Diabetes. *Clin Pract Pediatr Psychol* 2017;5(4):415-425. doi: 10.1037/cpp0000211.

17. Datye K, Bonnet K, Schlundt D, et al. Experiences of Adolescents and Emerging Adults Living With Type 1 Diabetes. *Diabetes Educ* 2019;45(2):194-202. doi:10.1177/0145721718825342

For peer review only - http://bmjopen.bmj.com/site/about/guidelines.xhtml

18. Balasanthiran A, O'shea T, Moodambail A, Woodcock T, Poots AJ, Stacey M, Vijayaraghavan S. Type 2 diabetes in children and young adults in East London: an alarmingly high prevalence. *Pract Diabetes* 2012;29(5):193-8a.

19. Sharpe D, Green E, Harden A, Freer R, Moodambail A, Towndrow S. 'It's my diabetes': Co-production in practice with young people in delivering a 'perfect' care pathway for diabetes. *Rese for All* 2018;2(2):289-303.

20. O'Brien BC, Harris IB, Beckman TJ, Reed DA, Cook DA. Standards for reporting qualitative research: a synthesis of recommendations. *Acad Med* 2014;89(9):1245-1251. doi:10.1097/ACM.0000000000000388

21. Braun V, Clarke V. What can "thematic analysis" offer health and wellbeing researchers? *Int J Qual Stud Health Well-being* 2014;9:26152. doi: 10.3402/qhw.v9.26152.

22. Deaux K, Reid A, Mizrahi K, et al. Parameters of social identity. *J Pers Soc Psychol* 1995;68(2):280.

23. Howard JA. Social psychology of identities. *Annu Rev Sociol* 2000;26(1):367-93. doi:10.1146/annurev.soc.26.1.367

24. Rose S, Paul C, Boyes A, et al. Stigma-related experiences in non-communicable respiratory diseases: A systematic review. *Chron Respir Dis* 2017;14(3):199-216. doi:10.1177/1479972316680847

25. Schabert J, Browne JL, Mosely K, et al. Social stigma in diabetes: a framework to understand a growing problem for an increasing epidemic. *Patient* 2013;6(1):1-10. doi:10.1007/s40271-012-0001-0

26. Smith LB, Terry A, Bollepalli S, et al. School-Based Management of Pediatric Type 1 Diabetes: Recommendations, Advances, and Gaps in Knowledge. *Curr Diab Rep* 2019;19(7):37. doi:10.1007/s11892-019-1158-x

For peer review only

For peer review only - http://bmjopen.bmj.com/site/about/guidelines.xhtml

27. Hinton D, Kirk S. Teachers' perspectives of supporting pupils with long-term health conditions in mainstream schools: a narrative review of the literature. *Health Soc Care Community* 2015;23(2):107-120. doi:10.1111/hsc.12104

28. Edwards D, Noyes J, Lowes L, et al. An ongoing struggle: a mixed-method systematic review of interventions, barriers and facilitators to achieving optimal self-care by children and young people with type 1 diabetes in educational settings. *BMC Pediatr* 2014;14:228. Published 2014 Sep 12. doi:10.1186/1471-2431-14-228

29. Hatherly K, Overland J, Smith L, et al. Providing optimal service delivery for children and adolescents with type 1 diabetes: a systematic review. *Pract Diab Int* 2009;26(4):154-9.

30. Kelo M, Martikainen M, Eriksson E. Self-care of school-age children with diabetes: an integrative review. *J Adv Nurs* 2011;67(10):2096-2108. doi:10.1111/j.1365-2648.2011.05682.x

31. Marks A, Wilson V, Crisp J. The management of type 1 diabetes in primary school: review of the literature. *Issues Compr Pediatr Nurs* 2013;36(1-2):98-119. doi:10.3109/01460862.2013.782079

32. Balfe M. The body projects of university students with type 1 diabetes. *Qual Health Res* 2009;19(1):128-139. doi:10.1177/1049732308328052

33. Sparapani Vde C, Borges AL, Dantas IR, et al. Children with Type 1 Diabetes Mellitus and their friends: the influence of this interaction in the management of the disease. *Rev Lat Am Enfermagem* 2012;20(1):117-125. doi:10.1590/s0104-11692012000100016

34. Banks GG, Berlin KS, Keenan ME, et al. How Peer Conflict Profiles and Socio-Demographic Factors Influence Type 1 Diabetes Adaptation. *J Pediatr Psychol* 2020;45(6):663-672. doi:10.1093/jpepsy/jsaa036

35. Salamon KS, Brouwer AM, Fox MM, et al. Experiencing type 2 diabetes mellitus: qualitative analysis of adolescents' concept of illness, adjustment, and motivation to

For peer review only - http://bmjopen.bmj.com/site/about/guidelines.xhtml

engage in self-care behaviors. *Diabetes Educ* 2012;38(4):543-551. doi:10.1177/0145721712445214

36. Sherifali D, Viscardi V, Bai JW, et al. Evaluating the Effect of a Diabetes Health Coach in Individuals with Type 2 Diabetes. *Can J Diabetes* 2016;40(1):84-94. doi:10.1016/j.jcjd.2015.10.006

37. Tsiouli E, Alexopoulos EC, Stefanaki C, et al. Effects of diabetes-related family stress on glycemic control in young patients with type 1 diabetes: Systematic review. *Can Fam Physician* 2013;59(2):143-149.

38. Dashiff C, Morrison S, Rowe J. Fathers of children and adolescents with diabetes: what do we know?. *J Pediatr Nurs* 2008;23(2):101-119. doi:10.1016/j.pedn.2007.08.007

39. Edwards D, Noyes J, Lowes L, Spencer LH, Gregory JW. An ongoing struggle: a mixed-method systematic review of interventions, barriers and facilitators to achieving optimal self-care by children and young people with type 1 diabetes in educational settings. *BMC Pediatr* 2014;14(1):1-27.

40. Garrett CJ, Ismail K, Fonagy P. Understanding developmental psychopathology in Type 1 diabetes through attachment, mentalisation and diabetes distress. *Clin Child Psychol Psychiatry* 2021;1359104521994640. doi: 10.1177/1359104521994640

41. DeCosta P, Grabowski D, Skinner TC. The psychosocial experience and needs of children newly diagnosed with type 1 diabetes from their own perspective: a systematic and narrative review. *Diabet Med* 2020;37(10):1640-52.

42. Butler AM, Hilliard ME, Titus C, Rodriguez E, Al-Gadi I, Cole-Lewis Y, Thompson D. Barriers and Facilitators to Involvement in Children's Diabetes Management Among Minority Parents. *J Pediatr Psychol* 2020;45(8):946-956. doi: 10.1093/jpepsy/jsz103

For peer review only - http://bmjopen.bmj.com/site/about/guidelines.xhtml

43. Salway S, Holman D, Lee C, McGowan V, Ben-Shlomo Y, Saxena S, Nazroo J. Transforming the health system for the UK's multiethnic population. *BMJ* 2020;368:m268. doi: 10.1136/bmj.m268

44. McGavock J, Wicklow B, Dart AB. Type 2 diabetes in youth is a disease of poverty. *Lancet* 2017;390(10105):1829. doi: 10.1016/S0140-6736(17)32461-3

45. Hilliard ME, Hagger V, Hendrieckx C, Anderson BJ, Trawley S, Jack MM, Pouwer F, et al. Strengths, Risk Factors, and Resilient Outcomes in Adolescents With Type 1 Diabetes: Results From Diabetes MILES Youth-Australia. *Diabetes Care* 2017;40(7):849-855. doi: 10.2337/dc16-2688

46. Lukács A, Mayer K, Sasvári P, Barkai L. Health-related quality of life of adolescents with type 1 diabetes in the context of resilience. *Pediatr Diabetes* 2018;19(8):1481-1486. doi: 10.1111/pedi.12769

For peer review only

For peer review only - http://bmjopen.bmj.com/site/about/guidelines.xhtml

Reporting checklist for qualitative study.

Based on the SRQR guidelines.

Instructions to authors

Complete this checklist by entering the page numbers from your manuscript where readers will find each of the items listed below.

Your article may not currently address all the items on the checklist. Please modify your text to include the missing information. If you are certain that an item does not apply, please write "n/a" and provide a short explanation.

Upload your completed checklist as an extra file when you submit to a journal.

In your methods section, say that you used the SRQRreporting guidelines, and cite them as:

O'Brien BC, Harris IB, Beckman TJ, Reed DA, Cook DA. Standards for reporting qualitative research: a synthesis of recommendations. Acad Med. 2014;89(9):1245-1251.

		Reporting Item	Page Number
**Title**			1
	#1	Concise description of the nature and topic of the study identifying the study as qualitative or indicating the approach (e.g. ethnography, grounded theory) or data collection methods (e.g. interview, focus group) is recommended	
**Abstract**			2
	#2	Summary of the key elements of the study using the abstract format of the intended publication; typically includes background, purpose, methods, results and conclusions	
**Introduction**			4
Problem formulation	#3	Description and signifcance of the problem / phenomenon studied: review of relevant theory and empirical work; problem statement	

For peer review only

Purpose or research question	#4	Purpose of the study and specific objectives or questions	5/6
**Methods**			6
Qualitative approach and research paradigm	#5	Qualitative approach (e.g. ethnography, grounded theory, case study, phenomenolgy, narrative research) and guiding theory if appropriate; identifying the research paradigm (e.g. postpositivist, constructivist / interpretivist) is also recommended; rationale. The rationale should briefly discuss the justification for choosing that theory, approach, method or technique rather than other options available; the assumptions and limitations implicit in those choices and how those choices influence study conclusions and transferability. As appropriate the rationale for several items might be discussed together.	
Researcher characteristics and reflexivity	#6	Researchers' characteristics that may influence the research, including personal attributes, qualifications / experience, relationship with participants, assumptions and / or presuppositions; potential or actual interaction between researchers' characteristics and the research questions, approach, methods, results and / or transferability	6
Context	#7	Setting / site and salient contextual factors; rationale	6
Sampling strategy	#8	How and why research participants, documents, or events were selected; criteria for deciding when no further sampling was necessary (e.g. sampling saturation); rationale	6
Ethical issues pertaining to human subjects	#9	Documentation of approval by an appropriate ethics review board and participant consent, or explanation for lack thereof; other confidentiality and data security issues	7

For peer review only

Data collection methods	#10	Types of data collected; details of data collection procedures including (as appropriate) start and stop dates of data collection and analysis, iterative process, triangulation of sources / methods, and modification of procedures in response to evolving study findings; rationale	6
Data collection instruments and technologies	#11	Description of instruments (e.g. interview guides, questionnaires) and devices (e.g. audio recorders) used for data collection; if / how the instruments(s) changed over the course of the study	7
Units of study	#12	Number and relevant characteristics of participants, documents, or events included in the study; level of participation (could be reported in results)	Supplementary form
Data processing	#13	Methods for processing data prior to and during analysis, including transcription, data entry, data management and security, verification of data integrity, data coding, and anonymisation / deidentification of excerpts	7
Data analysis	#14	Process by which inferences, themes, etc. were identified and developed, including the researchers involved in data analysis; usually references a specific paradigm or approach; rationale	7
Techniques to enhance trustworthiness	#15	Techniques to enhance trustworthiness and credibility of data analysis (e.g. member checking, audit trail, triangulation); rationale	7
**Results/findings**			
Syntheses and interpretation	#16	Main findings (e.g. interpretations, inferences, and themes); might include development of a theory or model, or integration with prior research or theory	7
Links to empirical data	#17	Evidence (e.g. quotes, field notes, text excerpts, photographs) to substantiate analytic findings	7-14

For peer review only

Discussion

Intergration with prior work, implications, transferability and contribution(s) to the field	#18	Short summary of main findings; explanation of how findings and conclusions connect to, support, elaborate on, or challenge conclusions of earlier scholarship; discussion of scope of application / generalizability; identification of unique contributions(s) to scholarship in a discipline or field	15
Limitations	#19	Trustworthiness and limitations of findings	16
**Other**			
Conflicts of interest	#20	Potential sources of influence of perceived influence on study conduct and conclusions; how these were managed	18
Funding	#21	Sources of funding and other support; role of funders in data collection, interpretation and reporting	18

None The SRQR checklist is distributed with permission of Wolters Kluwer © 2014 by the Association of American Medical Colleges. This checklist can be completed online using https://www.goodreports.org/, a tool made by the EQUATOR Network in collaboration with Penelope.ai

For peer review only
